# Flies and *Campylobacter* Infection of Broiler Flocks

**DOI:** 10.3201/eid1008.040129

**Published:** 2004-08

**Authors:** Birthe Hald, Henrik Skovgård, Dang Duong Bang, Karl Pedersen, Jens Dybdahl, Jørgen B. Jespersen, Mogens Madsen

**Affiliations:** * Danish Institute for Food and Veterinary Research, Århus, Denmark;; † Danish Institute of Agricultural Sciences, Lyngby, Denmark;; ‡ DACS A/S, Nr. Snede, Denmark

**Keywords:** Campylobacter, chickens, poultry, insects, flies, Diptera, zoonoses, epidemiology, food contamination, national health programs, dispatch

## Abstract

A total of 8.2% of flies caught outside a broiler house in Denmark had the potential to transmit *Campylobacter jejuni* to chickens, and hundreds of flies per day passed through the ventilation system into the broiler house. Our study suggests that flies may be an important source of *Campylobacter* infection of broiler flocks in summer.

Campylobacteriosis, caused by *Campylobacter jejuni*, is the most common foodborne infection in industrialized countries, where it causes millions of cases of illness every year (1). Chicken products are the food items most often reported to be the source of human campylobacteriosis (1). Thus, eliminating *Campylobacter* from broilers is important for the safety of the food supply for humans (2,3) and is a priority in animal industrial production and health programs. In spring 2003, a Danish program against foodborne *Campylobacter* infection was launched. The strategy addresses multiple steps in the food supply chain "from stable to table" but focuses on reducing the prevalence of *Campylobacter* in chicken production by having comprehensive hygiene barriers between broiler houses and the environment (4,5).

For unexplained reasons, *Campylobacter* infection cannot be controlled during summer. Even strict compliance with all biosecurity regulations has failed to control the infection. In August 2003 in Denmark, for example, 72.1% of broiler flocks were infected (Danish Zoonosis Centre, www.dfvf.dk), a situation that left selling of *Campylobacter*-contaminated chicken meat to consumers inevitable. Although similarity between *Campylobacter* isolates from broiler flocks and animals in the surrounding areas has been shown (6), the transmission routes are not understood, as no contact between broiler flocks and animals outside the broiler house takes place in closed production systems. However, indirect contact may be established by flies that take up *Campylobacter* as they forage on fresh animal feces. We show that *Campylobacter*-infected flies entered a broiler house in large numbers through the ventilation systems, which suggests that flies may be an important vector in summer.

## The Study

The number of flies transported by means of ventilation air into a broiler house in Denmark was counted from July 22, 2003, to July 28, 2003, and the *Campylobacter* carriage rate of flies captured in the environment of the broiler house was estimated. The study period was chosen because flies generally peak in activity and abundance in July to August in Denmark. In addition, chickens in the broiler house and the animals in the area around the broiler house (5 sheep, 4 horses, and 1 dog with 10 puppies) were tested for carriage of *Campylobacter*. DNA from all *C. jejuni* isolates was analyzed by pulsed-field gel electrophoresis (PFGE) to determine if strains from different animals were similar.

The broiler house (80 m x 15 m) was located at Universal Transverse Mercator Grid zone 32, East 564,137 m, North 6,294,759 m (http://mac.usgs.gov/mac/isb/pubs/factsheets/fs07701.html). The facility was negative-pressure ventilated through a total of 84 wall valves for air intake (16.5 cm x 52.5 cm) and 12 round chimneys (diameter 62 cm) for active air outlet through the roof. The house was emptied, and the chickens (n = 28,235) were slaughtered on July 29, 2003. Reports of local weather data from the Danish Meteorological Institute (www.dmi.dk) for that week were a maximum day temperature of 25.4°C, a minimum night temperature of 11.9°C, and days with bright sunshine with no wind or rain.

Flies were collected in polyester nets equipped at two wall inlets (one net at each end of the house) in the dynamic air flow measured (7) at a pressure of 21 Pa of influx ventilation air (speed 3.6 m/s, volume 1,213 m^3^/h per inlet valve). After the nets were harvested, flies were visually sorted from other insects and counted. The flies, identified primarily to the order *Diptera* and the families *Muscidae* and *Calliphoridae*, were counted; the count showed that 917 ± standard deviation (SD) (843.5–990.5) flies ([a + b]/2 x 84, with a and b representing the number of flies in the two nets) had entered the broiler house per day through the ventilation system, or approximately 1 fly per 2,700 m^3^ of ventilation air (1,213 m^3^/h x 84 x 24 h/917). For this specific broiler house, this amount equals approximately 30,000 flies per broiler cycle in the summer season. To estimate the possibility of roof inlets as entrance route for flies into houses with an air inlet in the roof, the fan of one chimney was switched off. This step generated inlet air in this chimney. The result showed 167 flies per day entered through this one chimney, or an average of 4.5 flies per 2,700 m^3^ air. The flies captured in the ventilation system were used for counting only, since they were dead and dried out at the time of harvest.

To determine the prevalence of *Campylobacter* in flies around the broiler house, a total of 96 flies were captured singly within a distance of 50 m from the house by rackets equipped with small disposable plastic bags. Captured flies were narcotized with CO_2_. After species or genus was determined, each fly was transferred with a pair of tweezers to live storage in a sterile plastic tube with 1 mL of saline. The tubes were kept in an insulated container, which was transported to the laboratory within 24 hours after capture.

For *Campylobacter* detection, each fly was macerated in a sterile mortar, suspended in 2 mL of 0.9% saline. The mixture was centrifuged at 15,870 x *g* for 7 min. The pellet was resuspended in 2 mL of Bolton broth (CM0983 with SR0183 and SR0048) (Oxoid, Basingstoke, UK) and vortexed before incubation for enrichment at 37°C for 24 h. After enrichment, the tube was again centrifuged, and 100 µL of the sample from each of 49 tubes was streaked onto modified cefoperazone charcoal deoxycholate agar (mCCDA) (blood-free agar base supplemented with CM739 + SR155) (Oxoid). These plates were incubated at 42°C for 48 h in a microaerobic atmosphere (6% O_2_, 6% CO_2_, 4% H_2_ in N_2_). The tubes from the remaining 47 samples were subjected to DNA analysis by a nested polymerase chain reaction (PCR) (8).

**Figure 1:**
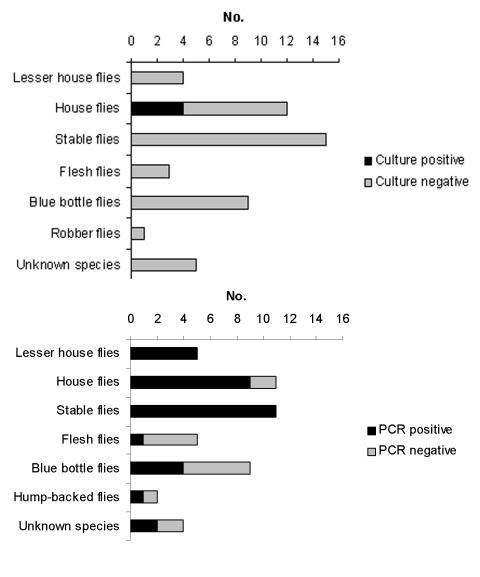
*Campylobacter*-infected flies captured around a broiler house in Denmark. Flies are grouped according to species. *Campylobacter*-positive and -negative by A) *Campylobacter* bacteriologic culture, and B) nested polymerase chain reaction.

The results of conventional culture showed that, of 49 flies tested, *C. jejuni* was isolated from 4 (8.2%) (Figure 1A); when a nested PCR was used, 33 (70.2%) of the 47 flies were *Campylobacter*-positive (Figure 1B). The species distribution according to the nested PCR when species-specific primers were used showed that, of 47 samples, 56.4% were positive with *C. jejuni* primers, 18.0% were positive with *C. coli* primers, and 25.6% were positive with *Campylobacter* species primers. Coinfection with *C. jejuni* and *C. coli* or *Campylobacter* spp. was found in six flies. However, PCR also detects nonculturable and dead *Campylobacter*. The reason for dividing the flies into two equal portions, one for conventional culture and the other for PCR, was to avoid reducing the assumed low number of *Campylobacter* on each fly.

Cloacal or rectal swab samples from 20 broilers, 5 sheep, and 4 horses were cultured on mCCDA agar, as described above. Rectal swabs from 11 dogs were streaked onto cefoperazone amphoricin teicoplanin (CAT) agar plates (blood-free agar base with CM739 and SR174) (Oxoid), and incubated at 42°C for 96 h. Subsequently, the swabs were tested for *Campylobacter* by our laboratory's routine PCR for feces (9). *C. jejuni* was isolated from 20 broilers and 4 sheep and *C. upsaliensis* from 11 dogs. One sheep and the four horses were culture-negative. However, all fecal swab samples were *Campylobacter*-positive by the routine PCR.

**Figure 2:**
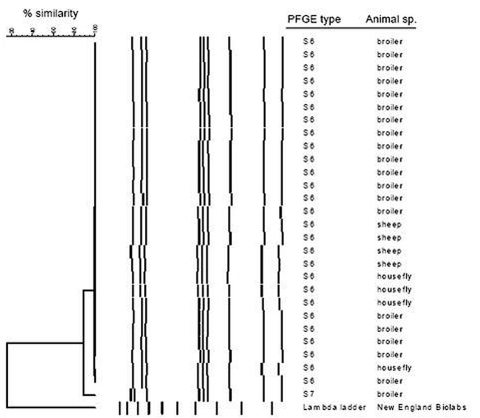
Dendrogram of pulsed-field gel electrophoresis types with *Sma*I of *Campylobacter jejuni* isolates from chickens in a Danish broiler house and from animals outside (flies and sheep).

A total of 28 *C. jejuni* isolates from 20 broilers, 4 flies, and 4 sheep were fingerprinted with PFGE with two different restriction enzymes, *Sma*I and *Kpn*I. With both enzymes, 27 of the isolates had an identical PFGE pattern (S6), whereas a single broiler isolate had a slightly different, but closely related pattern (S7), which probably was derived from the more prevalent pattern. The *Sma*I patterns are shown in Figure 2. Twenty-seven of 28 isolates from three animal sources, broilers, sheep, and flies, and from both inside and outside the broiler house, belonged to the same clone.

Under experimental conditions (10), flies are able to transmit *Campylobacter* among chickens. Moreover, a high prevalence of *Campylobacter*-infected flies captured in a broiler house has been found (11). However, no study has yet been able to demonstrate a significant role of flies captured in the houses for transmitting infection from flock to flock (5). Our results suggest that the potential of flies to transmit infection depends upon a current supply to the broiler house of *Campylobacter*-infected flies from the outside. Furthermore, the number of flies entering the broiler house must increase as the need for ventilation air increases as a consequence of the growth of chickens. Thus, the risk of introducing *Campylobacter* to the house increases with the age of the chickens.

## Conclusions

This study has demonstrated that flies pose a threat of *Campylobacter* infection, from which chickens currently are unprotected from April to October, when insects are in season in the Northern Hemisphere. We found that in July hundreds of flies per day passed through the ventilation system into a broiler house and that 8.2% of flies captured in the environment had the potential to transmit *C. jejuni* from outside animals to chickens in the broiler house. These results warrant further research on how to combat the summer peak of *Campylobacter* in broilers to improve the safety of the human food supply.
